# Lack of Additional Advanced Graduate Training by Orthopaedic Surgeons in Academic Practice: Current Employment and Recruitment Trends

**DOI:** 10.5435/JAAOSGlobal-D-20-00003

**Published:** 2020-05-18

**Authors:** Devon E. Anderson, Benjamin D. Kuhns, Shannon Kaupp, Edward M. Schwarz, Paul T. Rubery, Sandeep Mannava

**Affiliations:** From the Department of Orthopaedics and Rehabilitation (Dr. Anderson, Dr. Kuhns, Dr. Schwarz, Dr. Rubery, Dr. Mannava), University of Rochester, Rochester; and the State University of New York Upstate Medical University (Ms. Kaupp), Syracuse, NY.

## Abstract

**Background:**

Orthopaedic surgery is ever changing and depends on diverse technical and intellectual skill sets. The purpose of the current study was to evaluate the percentage of academic orthopaedic surgeons with additional graduate degrees in the United States.

**Methods:**

Data including advanced degree(s) (eg, PhD, MS, MBA, MPH, JD, and DVM), academic rank, leadership position, subspecialty, years since training completion, and sex were collected from websites for all academic orthopaedic surgery departments in the United States. Univariate analyses were performed to evaluate for differences in demographic data based on the advanced degree status. Data from the National Resident Matching Program (NRMP) were used to characterize graduate degree-holding US senior medical students who ranked orthopaedic surgery first relative to peers without additional advanced degrees and to applicants who ranked other specialties first.

**Results:**

Of 4,519 faculty at 175 academic orthopaedic surgery departments in the United States, 7.1% held a graduate degree in addition to a medical doctorate. There was no difference in the percentage of faculty who held departmental leadership positions (*P* = 0.62) or who were full professors (*P* = 0.66) based on holding an additional graduate degree. Of 678 US senior applicants who ranked orthopaedic surgery first and successfully matched into the specialty in 2018, 12.5% held an additional graduate degree and 1.3% were MD-PhDs. Orthopaedic surgery had the second lowest percentage of matched medical students with additional advanced degrees, which was significantly lower than the top 10 specialties (range 16.1% to 21.6%; *P* < 0.05). Orthopaedic surgery recruited 1.6% of all MD-PhD applicants in 2018.

**Discussion:**

Few academic orthopaedic surgery faculty and admitted orthopaedic residency candidates have additional graduate school training. The low percentage of orthopaedic faculty and trainees with additional advanced degrees relative to other specialties may represent a missed opportunity to recruit individuals with diverse skills to advance the field of orthopaedic surgery.

As the field of orthopaedic surgery evolves, practitioners are expected to gain familiarity with both the rapidly expanding literature and research methodology. This is especially true of trainees, who will shape the future of orthopaedic practice and innovation. Although many medical students gain research skills from mentors during undergraduate and medical school, no universal training requirements exist to provide all physicians with research competency. Orthopaedic surgery residents must meet necessary Accreditation Council for Graduate Medical Education (ACGME) requirements for research and publication; however, this limited exposure to research precludes mastering the skills that allow for continued, successful research. In addition, as both the business and regulatory aspects of medicine become more complex, most orthopaedic surgeons lack specific expertise and training in these fields.

Although not all physicians need to conduct high level research, business leadership, or legal advocacy, a subgroup of physicians have pursued these interests through formal graduate education. As an example, physician-scientist training programs, specifically the National Institutes of Health (NIH) funded Medical Scientist Training Programs (MSTP), and non-NIH funded MD-PhD training programs, are designed to give physicians specific skills to combine research with clinical practice. In comparison with orthopaedic surgery, other medical and surgical fields have more successfully recruited physician-scientists,^1-3^ who are formally trained in research methodology, funding procurement, and the peer-review process. These same skills are valued and emphasized in orthopaedic residency and academic practice, yet there has been a consistent lack of recruitment of graduate research trained medical students to orthopaedic surgery over the past 40 years.^4^ Similarly, individuals who pursued graduate degrees in public health, business, or law are well suited to navigate complex policy, business, and legal relationships in modern medical practice. Given their background, they have the ability to promote and protect the role of orthopaedic surgery in healthcare systems.^5^

Although low recruitment of MD-PhD physician scientists to orthopaedic surgery has been reported previously,^4,6,7^ the contribution of orthopaedic surgeons with any formal graduate education beyond a medical doctorate to the academic orthopaedic community remains unknown. The purpose of the current study was to quantify and characterize the proportion of academic faculty from all ACGME-accredited orthopaedic surgery residency programs in the United States who hold additional graduate degrees and to investigate the present recruitment trends of dual-degree students into orthopaedic surgery.

## Methods

### Data Acquisition

Demographic data for 4,519 orthopaedic surgery faculty from 175 academic orthopaedic surgery departments—defined as all programs with ACGME-accredited orthopaedic surgery residency training programs—in the United States in the 2017 to 2018 academic year were collected from publicly available websites for each department. Departments from allopathic, osteopathic, and uniformed services training programs were included. Data collected for each faculty member included advanced degree(s), academic rank (instructor, assistant professor, associate professor, and professor), leadership role (department chair, division chair, or residency program director), subspecialty, years since residency and/or fellowship completion, and sex.

Residency match data from 2007 to 2018 for US allopathic senior medical students were used with permission from publicly available *Charting Outcomes of the Match* reports, which are published by the National Residency Matching Program (NRMP).^8^ Specifically, match rates for applicants who ranked orthopaedic surgery first on the rank order list and held an additional graduate degree were evaluated relative to peers without additional advanced degrees and to applicants who ranked other specialties first.

### Statistical Analysis

Data were analyzed with JMP version 14.0 (Cary, NC). Study variables including sex, academic rank, subspecialty, and leadership were assessed with either percentages or means and standard deviations where appropriate. Categorical data were compared with a Student *t*-test or a 1-way analysis of variance based on the number of variables. Categorical variables and proportional analysis were analyzed with Pearson χ^2^ correlations. When multiple categorical variables (ie, PhD vs. MS vs. MBA) were compared, the Tukey-Kramer HSD was applied. Significance was determined by a *P* < 0.05.

## Results

**Figure 1:**
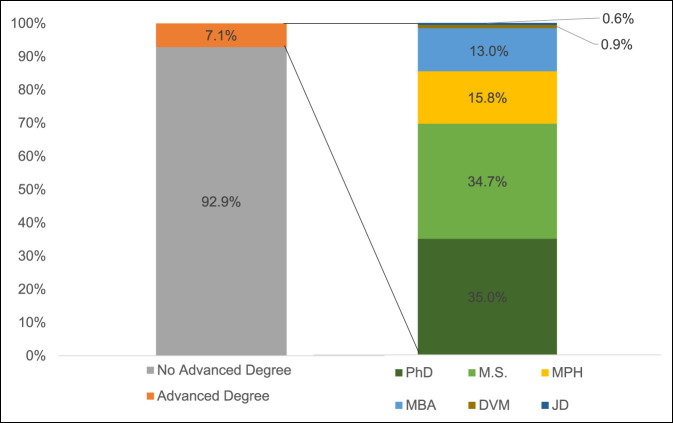
Graph demonstrating the distribution of academic orthopaedic faculty by additional advanced degree.

Of 4,519 orthopaedic surgeons in academic practice in the United States in 2017 to 2018, 320 (7.1%) held advanced degrees in addition to an MD or a DO medical degree, with 3 (0.07%) holding more than one. Of those with an additional graduate degree, 202 (63.1%) held master's and 118 (36.8%) held doctorate degrees. Distribution of advanced degrees by type was as follows: 113 (35.0%) PhD, 112 (34.7%) MS, 51 (15.8%) MPH, 42 (13.1%) MBA, three DVM (0.9%), and two JD (0.6%) (Figure 1).

Advanced degree distribution was evaluated based on sex, academic rank, orthopaedic subspecialty, leadership, and practice duration (Table 1). There were no proportionate sex differences associated with advanced degrees. There was a trend toward a lower proportion of female surgeons with PhDs (5/435; 1.1%) compared with male surgeons with PhDs (108/4,063; 3%; *P* = 0.06) and a lower percentage of female MD-PhDs (5/113; 4.4%) compared with female MDs (430/4,385; 9.8%; *P* < 0.001). Within each subspecialty, orthopaedic oncologists had a significantly higher proportion of MD-PhDs (13/211; 6.2%) compared with hand and upper extremity (4/314; 1.3%; *P* = 0.02), spine (10/552; 1.8%; *P* = 0.02), sports medicine (19/851; 2.2%; *P* = 0.04), and pediatric (7/478; 1.5%; *P* = 0.01) subspecialists. Arthroplasty surgeons comprised the greatest overall percentage of MD-PhDs (22/113; 19.5%).

**Table 1:** Univariate Analyses to Compare Distribution of Academic Orthopaedic Faculty With or Without an Additional Advanced Degree

Variable	Advanced Degree		Total Faculty	*P*
	No	Yes		
Sex				
Female	402 (92.4%)	33 (7.6%)	435	0.66
Male	3,797 (93.0%)	287 (7.0%)	4,084	
Not listed			0	
Academic rank				
Instructor	145 (92.4%)	12 (7.6%)	157	n.s
Assistant professor	1,346 (90.5%)	142 (9.5%)	1,488	
Associate professor	721 (92.8%)	56 (7.2%)	777	
Professor	753 (91.1%)	74 (8.9%)	827	
Not listed			1,270	
Subspecialty				
General	171 (94.0%)	11 (6.0%)	182	n.s
Pediatric	441 (92.3%)	37 (7.7%)	478	
Trauma	407 (92.1%)	35 (7.9%)	442	
Arthroplasty	586 (92.7%)	46 (7.3%)	632	
Shoulder/elbow	102 (92.8%)	8 (7.2%)	110	
Hand/upper extremity	592 (93.7%)	40 (6.3%)	632	
Foot and ankle	292 (93.0%)	22 (7.0%)	314	
Spine	510 (92.4%)	42 (7.6%)	552	
Sports medicine	801 (94.1%)	50 (5.9%)	851	
Oncology	187 (88.6%)	24 (11.3%)	211	
Not listed			115	
Leadership				
No	3,697 (93.0%)	279 (7.0%)	3,976	0.65
Yes	502 (92.4%)	41 (7.6%)	543	
Not listed			0	
Years in practice				
<10 years	1,137 (90.8%)	115 (9.2%)	1,252	**<0.0001**
>10 years	2,818 (94.1%)	178 (5.9%)	2,996	
Not listed			271	

n.s = No significant differences between the subspecialty or academic rank using the Tukey-Kramer HSD. Not listed indicates data not available on public faculty profile.

Academic orthopaedic faculty holding an additional advanced degree other than a PhD had been in practice a significantly shorter time than faculty not holding another advanced degree (*P* < 0.0001). This difference in shorter time in practice was also significant for faculty with a PhD relative to faculty without a PhD (*P* = 0.007). No difference was observed in the percentage of faculty with or without advanced degrees (*P* = 0.66), including PhDs (*P* = 0.99), who were full professors versus those who held lower academic ranks. Similarly, no difference was noted in the percentage of those in academic department leadership roles based on holding any additional advanced degree (*P* = 0.65) or a PhD (*P* = 0.45).

**Figure 2:**
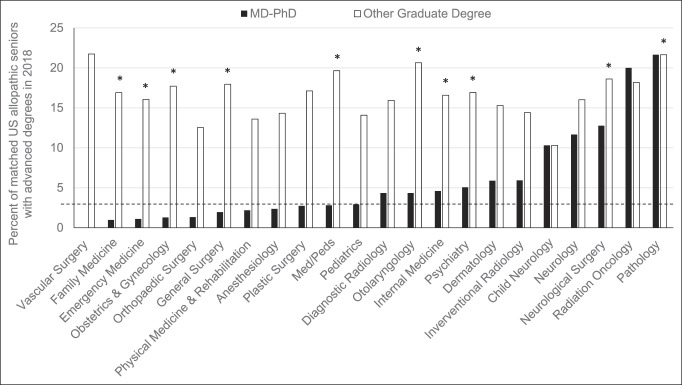
Graph demonstrating the representation of the percentage of all US senior applicants with an MD-PhD (black) or any additional graduate degree (white) who matched to a given specialty in 2018. Percentages for applicants with an MD-PhD (black) within a specific specialty that are above the dashed line were significantly greater than for orthopaedic surgery (*P* < 0.05). *Percentages for applicants with an additional advanced degree (white) within a specific specialty were significantly greater than for orthopaedic surgery (*P* < 0.05). Data derived from 2018 *Charting Outcomes of the Match* Report.^8^

**Figure 3:**
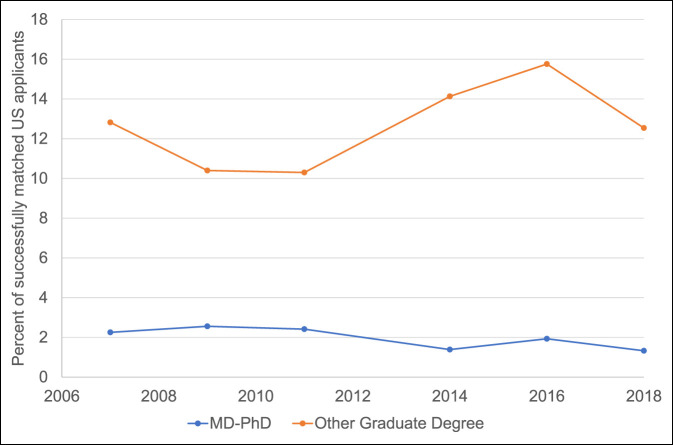
Graph demonstrating the representation of the percentage of US senior MD-PhD applicants who matched into orthopaedics from 2007 to 2018. Data derived from 2018 *Charting Outcomes of the Match* Report.^8^

**Figure 4:**
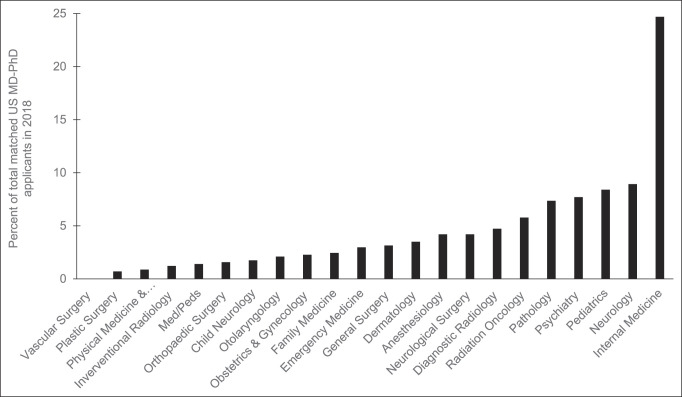
Graph demonstrating the representation of the percentage of all US senior MD-PhD applicants who matched to a given specialty in 2018. Data derived from 2018 *Charting Outcomes of the Match* Report.^8^

Among 678 total senior allopathic medical students in the United States who ranked orthopaedic surgery first and successfully matched into the specialty in 2018, 85 (12.5%) held additional graduate degrees, and nine (1.3%) held a PhD. Orthopaedic surgery had the fifth lowest percentage of successful applicants holding a PhD, which was significantly lower than 12 other specialties (*P* < 0.05; Figure 2, black bars). Vascular surgery recruited no MD-PhD applicants (0%), whereas pathology recruited the most (21.6%). The distribution of specific graduate degrees aside from PhDs is not reported. Orthopaedic surgery had the second lowest percentage of matched applicants who held any additional graduate degree aside from a PhD, which was significantly lower than 10 other specialties (*P* < 0.05; Figure 2, white bars). Vascular surgery had the highest percentage (21.7%) of successful applicants with additional advanced degrees, whereas pediatric neurology had the lowest (10.3%). The overall match rate for a US senior applicant to orthopaedic surgery in 2018 was 80.8%. For orthopaedic surgery, the 2018 match rate for applicants with additional graduate degrees or an MD-PhD was 76.9% and 75.0%, respectively. Since 2007, there has been an overall decline in the number of US senior medical students with a PhD who match into orthopaedic surgery, with an all-time low of 1.3% in 2018 (range 1.3% to 2.6%; *P* = 0.11; Figure 3). Over this same period from 2007 to 2018, the percentage of applicants with an additional graduate degree, other than a PhD, increased from 2011 to 2016 to a maximum of 15.8% but declined to 12.5% in 2018 (range 10.3% to 15.8%; *P* < 0.05; Figure 3). Of all graduating MD-PhD applicants to the 2018 residency match, 1.6% of these applicants ranked orthopaedic surgery first and successfully matched into the specialty (Figure 4).

## Discussion

To our knowledge, this is the first study to report the percentage of academic orthopaedic surgery faculty who hold a graduate degree in addition to a medical doctorate. The overall composition of orthopaedic surgery faculty with an additional graduate degree is 7.1%, which is remarkably low when considering the percentage of total residency applicants with additional advanced degrees (19.1%) and the number of dual-degree training programs in the United States. When compared with data reported by Clark et al in 2001,^7^ the percentage of orthopaedic MD-PhD faculty has remained stagnant from 2.0% in 2001 to 2.5% in 2018 despite a decrease in recruitment of MD-PhD students over the past decade.

The percentage of women orthopaedic faculty with additional advanced degrees (10.3%) was consistent with the overall percentage of women faculty in orthopaedic surgery (9.6%). Women represented 14.0% of orthopaedic surgery residents,^9^ and the percentage of women among orthopaedic faculty included in the 2016 AAMC Faculty Roster was reported as 17.8%.^9^ After review of the current database and the 2016 AAMC Faculty Roster, this difference in percentage of women is based on inclusion of nonclinical and research orthopaedic faculty in the AAMC database. Although 40.3% of all MD-PhD graduates are women,^10^ recruitment of women orthopaedic MD-PhDs is exceedingly low. Only five of 112 MD-PhD orthopaedic surgery faculty are women, comprising 0.1% of academic orthopaedic surgeons. Despite heightened awareness to the overall sex imbalance in orthopaedic surgery,^9-11^ we are unaware of efforts to recruit women who hold additional advanced degrees to orthopaedic surgery. Such recruitment could help balance disparities in musculoskeletal research and leadership.

A significant difference was observed in the average number of years in practice since the completion of postgraduate training; orthopaedic surgeons with an additional advanced degree were, on average, in practice four years less than peers without an additional advanced degree. From this data set, it was not possible to determine whether the additional degree was obtained through a combined degree-conferring program or whether the additional degree was obtained before medical school, during residency, or while in practice. We expect the overall career of a surgeon-scientist to be shorter based on age at completion of training. There was a significantly higher percentage of MD-PhD orthopaedic faculty with fellowship training in arthroplasty and oncology relative to the other subspecialties. This difference may represent the respective engineering and basic science content and increased research opportunities within those specialties. No difference was observed in the percentage of faculty with additional graduate degrees who held departmental leadership positions or who were full professors relative to their peers without an additional graduate degree. This was a surprising result because surgeon-scientists receive formal training during graduate school to attain metrics that have previously been associated with traditional academic success and promotion including the following: NIH funding, number of publications, Hirschberg index, leadership roles in specialty societies, and editorial board positions for top orthopaedic journals.^12^

Leaders in academic medicine—including department chairs and residency program directors—require skills distinct from clinical practice or research. Despite having 63 combined MD-MBA programs in the United States,^13^ only 42 academic orthopaedic faculty existed with an MBA. Few orthopaedic surgeons receive any formal business and leadership training; however, orthopaedic surgeons who seek leadership positions are expected to navigate complex hospital and medical systems while maintaining clinical practice, research, and teaching. To meet the need of formal business training for academic physicians, many business schools and professional societies now offer short courses in business topics.^5^ Similar to business acumen, an understanding of health policy, advocacy, and litigation is a valuable asset in modern medical practice, yet only two orthopaedic faculty in the US hold a JD despite 18 medical/law schools offering a MD-JD dual degree.^13^ Tapping into the current pool of residency applicants with dual MD-MBA or MD-JD degrees would afford orthopaedic surgery a potential pipeline of future business leaders and health policy advocates with formal training in skills and knowledge, otherwise not learned in medical training.

In accordance with the low percentage of orthopaedic faculty with additional graduate degrees, data from the 2018 residency match reveal that orthopaedic surgery has a lower percentage of matched applicants with additional graduate degrees than all other specialties, aside from pediatric neurology. MD-PhDs are separated in the aggregate data, which show a steady decrease in the percentage of successful MD-PhD applicants to orthopaedic surgery over the past decade to an all-time low in 2018. The career choices of MD-PhD students have gradually shifted over the course of MSTP funding to include a more diverse representation of specialties,^14^ yet the current data indicate that orthopaedic surgery is not included. Previously identified factors that may steer MD-PhD applicants from orthopaedic surgery may be related to the temporal investment required for surgical training, the individual student's personality and preferences, the training program/medical school, lack of mentorship in orthopaedics, or orthopaedic surgery as a field.^4,6,7^ In addition, MD-PhD students are not being recruited; holding a PhD is one the least influential factors in the residency application process according to a survey conducted of orthopaedic surgery residency program directors.^15^ This likely represents prioritization of clinical skills in the residency application process; however, the additional graduate degree does not come at a cost of reduced clinical acumen because these applicants have equivalent medical education to their peers. Based on 2016 data, orthopaedic surgery recruits the lowest percentage of MD-PhD applicants among all other competitive surgical subspecialties.^4^ Residency applicants who hold another graduate research degree reliably gain skills to produce high-quality research and to attain metrics of academic achievement, and orthopaedic departments should be actively pursuing these applicants to compete for these applicants with other surgical subspecialties.

Currently, all residents engage in research as required by the ACGME for program accreditation, which has the potential to shape the research scope and productivity of an orthopaedic department. Research productivity before residency, however, has been shown to be a positive predictor of research output during residency and beyond.^16-18^ Many programs have integrated a one-year research track for select residents. These programs require investment by the department, yet they do not lead to a definite increase in productivity after residency nor do they result in those residents entering academic careers.^19^ In comparison with MSTP-trained surgeon-scientists, physicians who participated in nondegree conferring postgraduate research programs have been less successful in eventually being awarded NIH grants and have fewer publications over the course of their careers.^20^ By not actively recruiting from the 19.1% of residency applicants with additional graduate training, including MD-PhD students, orthopaedic surgery may be discounting a significant cohort of future surgeon-scientists with the knowledge base and skill-set required to advance the field.

To our knowledge, this is the first study to investigate and report the prevalence of academic orthopaedic faculty with formal graduate education in addition to a medical doctorate. A limitation of this study is reliance on publicly available data to build a data set. This may lead to inconsistencies or inaccuracies. For example, we cannot guarantee that the publicly available website data were updated or included the appropriate graduate training record for each faculty member at a given time. Another limitation of this study was the restriction of this study to academic orthopaedic faculty. There are orthopaedic surgeons in private practice who hold additional advanced degrees, conduct meaningful research, and provide leadership in orthopaedics from scientific, business, and legal perspectives. Conversely, a subset of academic orthopaedic faculty exists with advanced degrees who do not conduct research or practically employ their graduate training. In the future, a similar analysis could be performed by assessing individual members within the American Academy of Orthopaedic Surgeons to characterize the prevalence of dual-degree faculty in private and academic practice, but this was beyond the scope and objective of the current study. Despite these limitations, it is valuable to appreciate the relatively small cohort of academic orthopaedic clinical faculty with additional advanced graduate degrees to increase awareness for future recruitment and benefit from the diverse and valuable skills attained with additional graduate training.

The data from the present study demonstrate that orthopaedic surgeons with additional advanced degrees are in the minority of both practicing academic surgeons and admitted residency applicants. This study is not intended to point out deficiencies in the current state of academic orthopaedic practice; instead, we seek to highlight a potential opportunity to harness the valuable skill sets of graduate-trained residency applicants to contribute to the future of orthopaedic surgery. Currently, orthopaedic surgery has few academic faculty with additional advanced degrees relative to the number of medical school graduates who have pursued additional graduate training and are being recruited by other surgical subspecialties. Lack of recruitment of dual-degree applicants represents a missed opportunity because orthopaedic surgeons with additional advanced graduate education have the potential to further diversify the field to best equip orthopaedics as a specialty poised to meet the research, business, and legal demands of modern medicine.

## References

[1] Brass LF Akabas MH Burnley LD Engman DM Wiley CA Andersen OS: Are MD–PhD programs meeting their goals? An analysis of career choices made by graduates of 24 MD–PhD programs. Acad Med 2010;85:1-10. 10.1097/ACM.0b013e3181d3ca17 PMC4441397 20186033

[2] Andriole DA Whelan AJ Jeffe DB: Characteristics and career intentions of the emerging MD/PhD workforce. J Am Med Assoc 2008;300:1165-1173. 10.1001/jama.300.10.1165 18780845

[3] Ahn J Man LX Wanderer J Bernstein J Iannotti JP: The future of the orthopaedic clinician-scientist. J Bone Joint Surg Am 2008;90:1794-1799. 18676927 10.2106/JBJS.G.00460

[4] Buckwalter JA Elkins JM: The scarcity of orthopaedic physician scientists. Iowa Orthop J 2017;37:219-224. 28852361 PMC5508265

[5] Emery SE: Filling the pipeline. J Bone Joint Surg Am 2017;99:e83-6. 28763421 10.2106/JBJS.17.00131

[6] Hurwitz SR Buckwalter JA: The orthopaedic surgeon scientist: An endangered species? J Orthop Res 1999;17:155-156. 10221830 10.1002/jor.1100170202

[7] Clark JM Hanel DP: The contribution of MD-PhD training to academic orthopaedic faculties. J Orthop Res 2001;19:505-510. 11518253 10.1016/S0736-0266(00)00051-6

[8] National Resident Matching Program: Charting Outcomes in the Match: U.S. Allopathic Seniors. Washington, DC, 2018:1-220.

[9] Chambers CC Ihnow SB Monroe EJ Suleiman LI: Women in orthopaedic surgery. J Bone Joint Surg Am 2018;100:e116-7. 30180066 10.2106/JBJS.17.01291

[10] Jeffe DB Andriole DA Wathington HD Tai RH: The emerging physician–scientist workforce. Acad Med 2014;89:1398-1407. 25006709 10.1097/ACM.0000000000000400 PMC4175019

[11] Rohde RS Wolf JM Adams JE: Where are the women in orthopaedic surgery? Clin Orthop Relat Res 2016;474:1950-1956. 27090259 10.1007/s11999-016-4827-y PMC4965367

[12] Namavar AA Loftin AH Khahera AS: Evaluation of US Orthopaedic Surgery Academic Centers based on measurements of academic achievement. J Am Acad Orthop Surgeons 2019;27:e118-e126. 10.5435/JAAOS-D-16-00536 30199475

[13] American Association of Medical Colleges: Medical Schools Offering a Combined Degree or Early Acceptance Programs: 2011-2012 Through 2015-2016. LCME Annual Medical School Questionnaire, Part II 2016.

[14] Andriole DA Jeffe DB: Predictors of full-time faculty appointment among MD–PhD program graduates: A national cohort study. Med Educ Online 2016;21:30941-30948. 27189673 10.3402/meo.v21.30941 PMC4870348

[15] Bernstein AD Jazrawi LM Elbeshbeshy B Valle CJD Zuckerman JD: Orthopaedic resident-selection criteria. J Bone Joint Surg Am 2002;84-A:2090-2096. 10.2106/00004623-200211000-00026 12429773

[16] Spitzer AB Gage MJ Looze CA Walsh M Zuckerman JD Egol KA: Factors associated with successful performance in an orthopaedic surgery residency. J Bone Joint Surg 2009;91:2750-2755. 19884456 10.2106/JBJS.H.01243

[17] Kreitz T Verma S Adan A Verma K: Factors predictive of orthopaedic in-training examination performance and research productivity among orthopaedic residents. J Am Acad Orthop Surgeons 2019;27:e286-e292. 10.5435/JAAOS-D-17-00257 30252788

[18] Namdari S Jani S Baldwin K Mehta S: What is the relationship between number of publications during orthopaedic residency and selection of an academic career? J Bone Joint Surg Am 2013;95:e45-6. 23553308 10.2106/JBJS.J.00516

[19] Brandt AM Rettig SA Kale NK Zuckerman JD Egol KA: Can a clinician-scientist training program develop academic orthopaedic surgeons? One program's thirty-year experience. J Surg Educ 2018;75:1039-1044. 29102560 10.1016/j.jsurg.2017.10.003

[20] Kosik RO Tran DT Fan APC: Physician scientist training in the United States. Eval Health Professions 2014;39:3-20. 10.1177/0163278714527290 24686746

